# Intra-hospital transport of newborn infants dataset

**DOI:** 10.1016/j.dib.2021.107510

**Published:** 2021-10-29

**Authors:** Romaine Delacrétaz, Céline J. Fischer Fumeaux, Corinne Stadelmann, Adriana Rodriguez Trejo, Alice Destaillats, Eric Giannoni

**Affiliations:** a Department of Pediatrics, eHnv Yverdon-les-Bains, Switzerland; b Department Mother-Woman-Child, clinic of Neonatology, Lausanne University Hospital and University of Lausanne, Switzerland; c Department Mother-Woman-Child, Mother-Child Research Unit, Lausanne University Hospital and University of Lausanne, Switzerland

**Keywords:** Neonate, Infant, Transport, Intra-hospital, Adverse event, Safety, Intensive care

## Abstract

This article presents a dataset on intra-hospital transport of newborn infants. We collected prospectively data from patients hospitalized between 1.6.2015 and 31.5.2017 at the tertiary care neonatal unit of the University Hospital of Lausanne, Switzerland. An intra-hospital transport was defined as a transport for a diagnostic or a therapeutic intervention outside the neonatal unit, but within the hospital. Healthcare professionals present during the transport collected data in a case report form. We obtained additional data from electronic medical charts and through the clinical information system Metavision®.

We recorded information on patients’ demographics and clinical characteristics, transports (indication, date, duration, destination, number and type of staff involved, medical devices and treatments), adverse events and interventions. Heart rate, peripheral oxygen saturation and fraction of inspired oxygen were recorded within 5 min before and after the transport, with an additional measure during transport for patients that had continuous monitoring of vital signs.

This dataset will be of use to clinicians, researchers and policy makers, to inform clinical practice, for benchmarking, and for the development of future guidelines. These data have been further analyzed and interpreted in the article “Adverse events and associated factors during intra-hospital transport of newborn infants” (Delacrétaz et al, 2021).

**Specifications Table**

**Table:** 

Subject	Health and medical sciences
Specific subject area	Neonatology
Type of data	Table Figure
How data were acquired	Paper based case report form Metavision® patient clinical information system (iMDsoft, Massachusetts, USA) Soarian® electronic medical chart (Siemens, Munich, Germany)
Data format	Raw Analyzed Filtered
Parameters for data collection	Data was collected on infants hospitalized in the tertiary care neonatal unit of the University Hospital of Lausanne, Switzerland, who underwent intra-hospital transport between June 1, 2015 and May 31, 2017. We defined an intra-hospital transport as a transport outside the neonatal unit, but within the hospital for a diagnostic or a therapeutic intervention.
Description of data collection	Data on patient demographics, clinical characteristics, transports, vital signs and adverse events was collected by healthcare professionals present during transport through a case report form. Additional data was extracted from electronic medical charts and the clinical information system of the neonatal unit.
Data source location	Institution: Lausanne University Hospital City/Town/Region: Lausanne, Vaud Country: Switzerland
Data accessibility	Repository name: Zenodo Data identification number: 10.5281/zenodo.5547732
Related research article	**Co-submission:** R. Delacrétaz, C.J. Fischer Fumeaux, C. Stadelmann, A. Rodriguez Trejo, A. Destaillats, E. Giannoni. Adverse events and associated factors during intra-hospital transport of newborn infants. The Journal of Pediatrics. 2021 Sep 1;S0022-3476(21)00859-3. doi:10.1016/j.jpeds.2021.08.074.

**Value of the Data**

• Intra-hospital transport of patients is frequently required to perform diagnostic or therapeutic procedures. The risks of intra-hospital transport are well documented in adults and children, but literature concerning newborns is scarce. This large dataset provides information on demographics, clinical characteristics and adverse events of newborns who underwent intra-hospital transport.
• Clinicians, researchers and policy makes involved in the fields of neonatology, critical care, hospital pediatrics and anesthesia/perioperative care will benefit from these data.
• The data may help clinicians for benchmarking and quality improvement initiatives, and researchers for comparisons with their own data on intra-hospital transport of neonates and future meta-analyses. The data may provide insights to policy makers, for the development of guidelines on intra-hospital transport of neonates.

## 1 Data Description

The dataset [TRI_database.xslx; TRI_codebook.pdf] provides information in 990 intra-hospital transports performed in 293 infants. Baseline demographics, clinical characteristics, characteristics of transports, adverse events that occurred during transports and interventions are documented.

Table 1 describes the main characteristics of the 990 transports that were included in the study, and the 412 transports that could not be included due to incomplete documentation.

**Table 1:** Main characteristics of the transports that could and could not be included in the study.

	Included	Not Included
Transports, *n* (%)	990 (71)	412 (29)
Female gender, n (%)	354 (36)	126 (31)
Median gestational age, weeks (Q1-Q3)	38 (34-39)	38 (34-40)
Median birthweight, g (Q1-Q3)	2478 (1451-3200)	2770 (1620-3300)
Median postnatal age at transport, days (Q1-Q3)	13 (5-44)	12 (5-39)

Descriptive statistics were based on the number of transports that could, or could not be included due to the lack of documentation; 293 patients were included, and 74 patients could not be included.

**Fig. 1:**
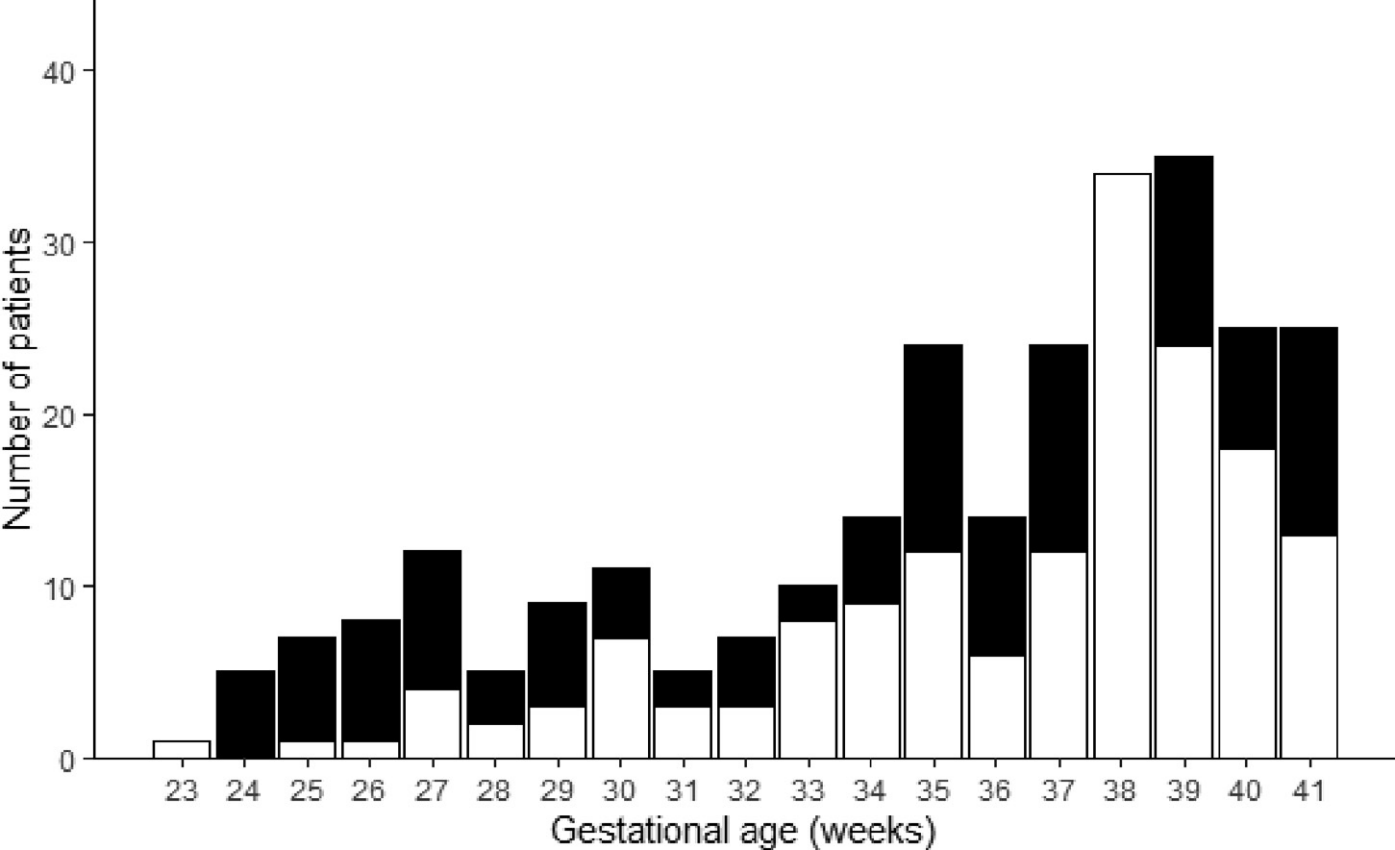
Gestational age of transported patients Gestational age of patients who had no adverse events during transport (white bars) and patients who had at least one transport complicated by an adverse event (black bars).

**Fig. 2:**
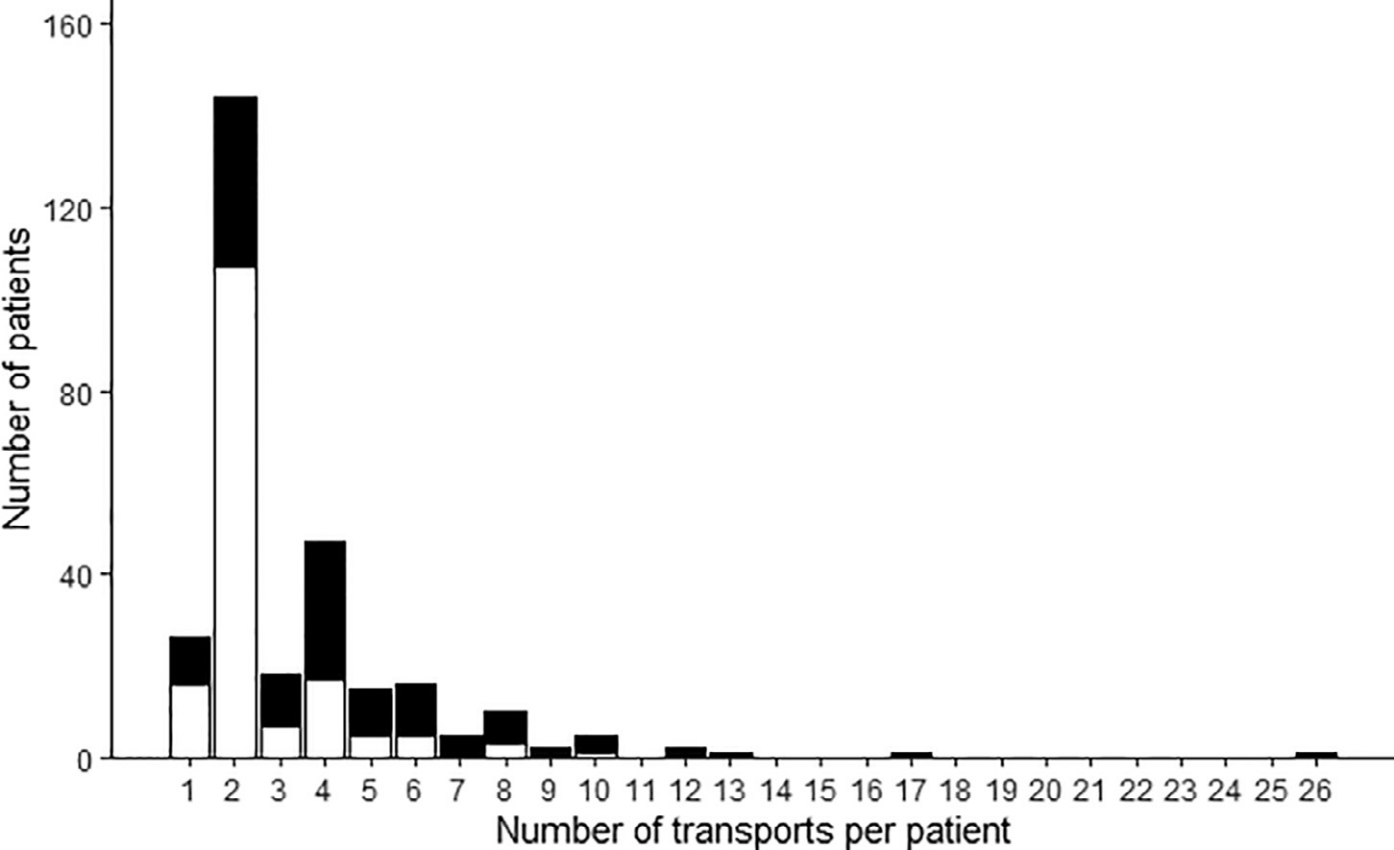
Number of transports per patient Number of transports per patient for patients who had no adverse events during transport (white bars) and for patients who had at least one transport complicated by an adverse event (black bars).

The median gestational age of the transported newborns was 38 weeks (Q1-Q3 34-39 weeks). The gestational age distribution is presented in Fig. 1 and Table 2. The median number of transports per patient was 2 (Q1-Q3 2-4). A histogram with the number of transports per patient is presented in Fig. 2.

**Table 2:** Number of transported patients for each gestational age group.

Gestational age groups	Number of patients (%)
Term newborns ≥ 37 weeks	161 (55)
Preterm newborns 34–36 ^6/7^	52 (18)
Preterm newborns 32,33 ^6/7^	17 (6)
Preterm newborns 28–31 ^6/7^	30 (10)
Preterm newborns 23–27 ^6/7^	33 (11)

Patients were transported in incubators (310/990, 31%), strollers (301/990, 30%), radiant warmers (136/990, 14%), Nomag®MR Diagnostics Incubator System (127/990, 13%), bed/cribs (94/990, 9%), and on the mother (2/990, 0.2%). The main characteristics of the transports with different vehicles are presented in Table 3.

**Table 3:** Main characteristics of transports with different vehicles.

Variables	Incubator n = 310	Stroller n = 301	Radiant warmer n = 136	Nomag® n = 127	Crib/bed n = 94	Other^a^ n = 2	*P* value^b^
Median postnatal age at the time of transport, days (Q1-Q3)	12 (4–40)	13 (7–53)	7 (2–29)	13 (7–32)	24 (9–-75)	90 (90–90)	0.02
Median weight at the time of transport, g (Q1-Q3)	2710 (1800–3320)	3060 (2510–3600)	3170 (2760–3550)	2810 (2190–3340)	3150 (2780–3780)	3720 (3720–3720)	< 0.001
Reason for transport:							
Magnetic resonance imaging, n (%)	28 (9)	92 (31)	22 (16)	125 (98)	7 (7)	0 (0)	< 0.001
Ultrasound, n (%)	24 (8)	111 (37)	11 (8)	2 (2)	34 (36)	0 (0)	
Surgery, n (%)	81 (26)	2 (1)	24 (18)	0 (0)	9 (10)	0 (0)	
Return from surgery, n (%)	68 (22)	2 (1)	15 (11)	0 (0)	4 (4)	0 (0)	
Bronchoscopy, n (%)	47 (15)	8 (3)	14 (10)	0 (0)	6 (6)	0 (0)	
Computerized tomography, n (%)	26 (8)	10 (3)	22 (16)	0 (0)	2 (2)	0 (0)	
Other^c^, n (%)	34 (11)	74 (25)	27 (20)	0 (0)	32 (34)	2 (100)	
Median duration of transport, min (Q1-Q3)	10 (10–15)	10 (7–10)	10 (10–15)	10 (7–13)	10 (7–10)	10 (9–10)	< 0.001
Median number of caregivers present during transport (Q1-Q3)	2 (2-2)	1 (1-2)	2 (2-3)	2 (2-2)	1 (1-2)	2 (2-2)	< 0.001
Respiratory support							< 0.001
Invasive ventilation, n (%)	108 (35)	0 (0)	39 (29)	22 (18)	0 (0)	0 (0)	
Non-invasive ventilation, n (%)	52 (17)	16 (5)	14 (10)	25 (20)	10 (11)	0 (0)	
Nasal cannula, n (%)	23 (7)	27 (9)	18 (13)	10 (8)	11 (12)	0 (0)	
Vascular access							
Peripheral venous catheter, n (%)	200 (65)	82 (27)	82 (60)	68 (54)	28 (30)	0 (0)	< 0.001
Central venous catheter^d^, n (%)	185 (60)	34 (11)	92 (68)	38 (30)	11 (12)	0 (0)	< 0.001
Arterial catheter^e^, n (%)	51 (17)	0 (0)	35 (26)	6 (5)	0 (0)	0 (0)	< 0.001
Gastric tube, n (%)	257 (83)	145 (48)	108 (79)	85 (67)	59 (63)	2 (100)	< 0.001
Bladder catheter, n (%)	38 (12)	4 (1)	21 (15)	6 (5)	2 (2)	0 (0)	< 0.001
Other medical device^f^, n (%)	9 (3)	0 (0)	3 (2)	0 (0)	1 (1)	0 (0)	0.03
Vasoactive drugs^g^, n (%)	32 (10)	0 (0)	22 (16)	2 (2)	0 (0)	0 (0)	< 0.001
Sedative and analgesics^h^, n (%)	103 (33)	4 (1)	44 (32)	27 (21)	1 (1)	0 (0)	< 0.001
Adverse events							< 0.001
No adverse event, n (%)	207 (67)	263 (87)	93 (68)	93 (73)	72 (77)	2 (100)	
Adverse event with no harm, n (%)	76 (25)	34 (11)	35 (26)	32 (25)	22 (23)	0 (0)	
Adverse event with mild harm, n (%)	25 (8)	4 (1)	7 (5)	1 (1)	0 (0)	0 (0)	
Adverse event with moderate harm, n (%)	2 (1)	0 (0)	1 (1)	1 (1)	0 (0)	0 (0)	

a Transport in the mother's arms.

b P values from Chi-squared test for categorical variables, and from analysis of variance for continuous variables.

c Including gastro-intestinal contrast studies, voiding cystourethrograms and other indications.

d Including umbilical venous catheters, peripherally inserted central catheters and other central venous catheters.

e Including umbilical and peripheral arterial catheters.

f Including peritoneal drainage, chest tube, and colostomy.

g Continuous infusion of catecholamines and/or prostaglandins.

h Continuous infusion only.

Potential predictors of adverse events during transports were analysed using a Generalized Estimating Equation, Table 4.

**Table 4:** Generalized Estimating Equations parameter estimates.

Parameter	Estimate	Standard error	95% Confidence limits		Z score	Pr > |Z|
Intercept	0.6486	1.3169	−1.9325	3.2297	0.49	0.6223
Gestational age	−0.0029	0.0422	−0.0855	0.0797	−0.07	0.9453
Birthweight	−0.0002	0.0002	−0.0007	0.0002	−1.04	0.2993
Postnatal age	0.0046	0.0027	−0.0007	0.0099	1.70	0.0889
Reason for transport	−0.0011	0.0031	−0.0072	0.0049	−0.37	0.7130
Duration of transport	0.0427	0.0167	0.0099	0.0755	2.56	0.0106
Number of caregivers present during transport	0.2058	0.1674	−0.1224	0.5339	1.23	0.2190
Respiratory support	0.1419	0.0928	−0.0400	0.3239	1.53	0.1262
Peripheral venous catheter	−0.1756	0.2135	−0.5941	0.2429	−0.82	0.4108
Central venous catheter^a^	−0.9313	0.2477	−1.4167	−0.4458	−3.76	0.0002
Arterial catheter^b^	0.5895	0.4191	−0.2319	1.4108	1.41	0.1596
Gastric tube^c^	0.3549	0.2495	−0.1341	0.8439	1.42	0.1549
Bladder catheter	−0.4892	0.3465	−1.1682	0.1899	−1.41	0.1580
Other medical device^d^	−0.7355	0.7256	−2.1577	0.6867	−1.01	0.3108
Vasoactive drugs^e^	−1.0015	0.3766	−1.7397	−0.2634	−2.66	0.0078
Sedative and analgesics	−0.0913	0.2593	−0.5995	0.4169	−0.35	0.7248
Transport vehicle	−0.0671	0.0737	−0.2115	0.0773	−0.91	0.3624

a Including umbilical venous catheters, peripherally inserted central catheters and other central venous catheters.

b Including umbilical and peripheral arterial catheters.

c Includes gastric and duodenal tubesd.

d Including peritoneal drainage, chest tube, and colostomy.

e Continuous infusion of catecholamines and/or prostaglandins.

Heart rate, peripheral oxygen saturation (SpO2), fraction of inspired oxygen (FiO2) and SpO2/FiO2 ratios before, during and after 169 transports of mechanically ventilated patients are presented in Table 5. Difference in heart rate, SpO2, FiO2 and SpO2/FiO2 ratios before and after transport are shown in Table 6.

**Table 5:** Vital signs in transports of mechanically ventilated patients.

	All transports*n* = 169	Transports with adverse event*n* = 75	Patients without adverse event*n* = 94	*P* value^a^
Median heart rate before transport, beats/min (Q1-Q3)	142 (130–157)	146 (125–160)	142 (131–156)	0.44
Median heart rate during transport, beats/min (Q1-Q3)	142 (129–158)	142 (130–165)	143 (129–155)	0.44
Median heart rate after transport, beats/min (Q1-Q3)	144 (130–156)	143 (123–165)	144 (133–153)	0.54
Median SpO2 before transport, % (Q1-Q3)	97 (94–99)	96 (94–99)	97 (95–99)	0.52
Median SpO2 during transport, % (Q1-Q3)	96 (94–98)	95 (93–98)	96 (95–98)	< 0.01
Median SpO2 after transport, % (Q1-Q3)	97 (95–98)	97 (93–98)	97 (95–98)	0.02
Median FiO2 before transport (Q1-Q3)	0.28 (0.21–0.39)	0.30 (0.22–0.45)	0.25 (0.21–0.35)	< 0.01
Median FiO2 during transport (Q1-Q3)	0.25 (0.21–0.35)	0.28 (0.23–0.38)	0.25 (0.21–0.30)	0.02
Median FiO2 after transport (Q1-Q3)	0.25 (0.21–0.35)	0.30 (0.23–0.39)	0.24 (0.21–0.30)	0.03
Median SpO2/FiO2 before transport (Q1-Q3)	355 (249–443)	322 (208–421)	379 (275–454)	0.02
Median SpO2/FiO2 during transport (Q1-Q3)	373 (277–433)	327 (235–392)	392 (313–452)	0.01
Median SpO2/FiO2 after transport (Q1-Q3)	373 (271–444)	327 (247–400)	400 (310–457)	< 0.01

a P values from pairwise tests (generalized linear mixed models).

**Table 6:** Difference in heart rate, oxygen saturation, and fraction of inspired oxygen before and after transport in mechanically ventilated patients.

	All transports^a^*n* = 169	Transports with adverse event^a^*n* = 75	Patients without adverse event^a^*n* = 94	P value^b^
Heart rate, beats/min	0 (−6;5)	1 (−7;6)	−1 (−5;5)	0.71
SpO2, %	0 (−1;1)	0 (−2;2)	0 (−1;1)	0.48
FiO2	0 (−0.04;0)	0 (−0.05;0)	0 (−0.03;0)	0.89
SpO2/FiO2	4 (−5.2;45.7)	4 (−11.4;57.7)	4 (−4.5;37.4)	0.61

a Values recorded before transport are subtracted from those recorded after transport. The median difference is reported as median (Q1;Q3).

b P values from pairwise tests (generalized linear mixed models).

The data have been further analyzed and interpreted in the article “Adverse events and associated factors during intra-hospital transport of newborn infants” [1].

## 2 Experimental Design, Materials and Methods

This prospective observational study was conducted in the medical and surgical tertiary care neonatal intensive care unit of the University Hospital of Lausanne, Switzerland. This 40-bed neonatal unit has 12 intensive care beds, 16 intermediate care beds and 12 specialized care beds.

Infants hospitalized in the neonatal unit were eligible for the study if they had been transported within the hospital between June 1, 2015 and May 31, 2017. We defined an intra-hospital transport as a transport outside the neonatal unit, but within the hospital for a diagnostic or therapeutic procedure. For patients who had undergone multiple transports, we considered each transport as a separate event. We excluded transports from the delivery room to the neonatal unit, and transports by ambulance and helicopter.

The nursing and medical staff of the neonatal unit performed all transports. Specific training on transport procedures and equipment is provided to nurses and physicians working in the neonatal unit, who perform both intra- and inter-hospital transports. For each transport, healthcare providers in charge chose on an individual basis which equipment, number and type of staff should be implicated.

Nurses and physicians present during the transport recorded data on a case report form. They collected data on the indication of the transport, duration, departure location and destination, number and type of healthcare providers involved, medical devices and treatments, adverse events and interventions. They recorded heart rate, peripheral oxygen saturation (SpO2) and fraction of inspired oxygen (FiO2) within 5 min before and after the transport, with an additional measurement during transport for patients with continuous monitoring of vital signs. We extracted data on the patients’ demographics (gestational age, birthweight, gender, postnatal age) and clinical characteristics (Apgar scores, umbilical cord arterial and venous pH, reason for admission in the neonatal unit, duration of hospital stay, mortality) from electronic medical records and from the patients’ clinical information system Metavision® (iMDsoft, Massachusetts, USA) [2,3].

We defined adverse events as any event considered by healthcare providers as a danger for the health of the infant, or by vital signs that had values outside the following reference ranges. We defined desaturation as a SpO2 below 85% in preterm infants (i.e. infants born before 37 weeks of gestation), and a SpO2 under 92% in term infants (i.e. infants born at a gestational age above 36 6/7 weeks of gestation). We defined hypothermia as a temperature below 36 °C, and hyperthermia as a temperature above 38 °C. We defined bradycardia as a heart rate under 90/min in preterm infants, and under 80/min for term infants. We defined tachycardia as a heart rate above 180/min. We defined hypotension as a mean arterial blood pressure less than the corrected or postmenstrual age, and hypertension as a systolic blood pressure greater than 95 mmHg in term infants, and greater than 85 mmHg in preterm infants [4], [5], [6], [7]. A complicated transport was defined as a transport with one or several adverse events.

We classified every complicated transport based on the level of harm according to the definitions of the World Health Organization (WHO) [8].

(1) No harm: the patient outcome is not symptomatic, or no symptoms are identified, and no treatment is required.
(2) Mild harm: the patient outcome is symptomatic, the symptoms are mild, the loss of function or harm is minimal or intermediate but short term, and no or minimal intervention (for example additional observation, investigation, review or minor treatment) is required.
(3) Moderate harm: the patient outcome is symptomatic, requiring intervention (for example additional operative procedure or medical treatment), an increased length of stay, or causing permanent or long-term harm or loss of function.
(4) Severe harm: the patient outcome is symptomatic, requiring life-saving intervention or major surgical/medical intervention, shortening life expectancy or causing major permanent or long-term harm or loss of function.
(5) Death: on balance of probabilities, death was caused or brought forward in the short term by the incident.

Four investigators (RD, CS, CF, EG) evaluated the severity of each adverse event. Each investigator independently reviewed each transport in which one or more adverse event(s) occurred and rated the level of harm according to the WHO classification [8]. Cases that received a discordant rating were discussed by the investigators in a focus group. The following consensus was reached: we considered abnormal blood pressure, heart rate and oxygen saturation, apnoea, neurological symptoms, hyperthermia and equipment problems as no harm if they resolved without treatment; we considered accidental hypothermia (34.5-35.9°C), hyperthermia, abnormal heart rate and blood pressure, and neurological symptoms as mild harm if they responded to simple medical therapy; we considered desaturation responding to manual ventilation and accidental hypothermia below 34.5°C as moderate harm after reviewing the cases in detail.

We presented descriptive statistics as absolute and relative frequencies for categorical variables, and as median, first and third quartiles (Q1–Q3) for continuous variables. For continuous variables, we analyzed differences between the groups with and without adverse events with parametric tests (t-test for normally distributed data) or non-parametric tests (Wilcoxon rank-sum test). We performed analysis of variance for comparisons between multiple groups. For categorical data, Pearson Chi-squared tests (or Fisher exact test when expected cell frequencies were less than 5) were utilized. We used a Generalized Estimating Equation to analyze potential predictors of adverse events during transports taking into account the correlation present in the data (multiple transports for the same patient). We measured vital signs before, during and after transport. We used generalized linear mixed models in order to evaluate a possible effect of time on vital signs, and possible differences between groups with and without adverse events. These models analyzed differences at time points during and after transport from baseline (before transport), within each complication group, and comparisons between groups at each time point, with adjustment for all comparisons. We used two-sided paired t-tests, and defined the statistical significance at *P* < 0.05. We conducted statistical analyses with R version 4.0.2 (R Foundation for Statistical Computing, Vienna, Austria).

## Ethics Statement

The study was approved by the Cantonal Ethics Committee of Vaud (Lausanne, Switzerland), protocol 28/15. The need for informed consent was waived due to the observational nature of the study. The research has been carried out in accordance with The Code of Ethics of the World Medical Association (Declaration of Helsinki).

## CRediT authorship contribution statement

**Romaine Delacrétaz:** Conceptualization, Methodology, Data curation, Investigation, Formal analysis, Writing – review & editing. **Céline J. Fischer Fumeaux:** Conceptualization, Methodology, Formal analysis, Writing – review & editing. **Corinne Stadelmann:** Conceptualization, Methodology, Resources, Writing – review & editing. **Adriana Rodriguez Trejo:** Formal analysis, Writing – review & editing. **Alice Destaillats:** Formal analysis, Writing – review & editing. **Eric Giannoni:** Conceptualization, Methodology, Data curation, Investigation, Formal analysis, Writing – original draft, Supervision.

## Declaration of Competing Interest

The authors declare that they have no known competing financial interests or personal relationships which have or could be perceived to have influenced the work reported in this article.

## Appendix Supplementary materials

Image, application 1

Image, application 2
