# Supplementary material for: Intra-hospital transport of newborn infants dataset
Source: Data Brief. 2021 Oct 29;39:107510. doi: 10.1016/j.dib.2021.107510 (PMC8572860; doi:10.1016/j.dib.2021.107510)
Supplement: Supplementary file 1 [file mmc1.pdf]

Codebook TRI database

Variable	Definition
Id_transp	Transport unique identification number
Id_patient	Patient unique identification number
Id_return	1 = outward transport ; 2 = return transport
pairing	Pairing with outward or return transport
sex	1 = female ; 2 = male
ga	Gestational age (weeks)
bw	Birthweight (grams)
admission	Reason for admission in the neonatal unit :<br>1. Prematurity ; 2. Malformation ; 3. Respiratory distress ; 4. Asphyxia/seizures ; 5. Infection ; 6. Other
apg_1	Apgar score at 1 minute
apg_5	Apgar score at 5 minutes
apg_10	Apgar score at 10 minutes
pH_a	Umbilical cord arterial pH
pH_v	Umbilical cord venous pH
dur_hosp	Duration of hospital stay (days)
death	Death before discharge
indic	Reason for transport:<br>1. Surgery; 2. Return from surgery; 3. Magnetic resonance imaging; 4. Computerized tomography; 5. Bronchoscopy; 6. Ultrasound; 88. Other
weight	Weight at transport (grams)
pna	Postnatal age at transport (days)
dep	Departure:<br>1. Neonatal Unit, intensive care; 2. Neonatal Unit, intermediate care; 3. Neonatal unit, specialized care; 4. Operating room; 5. Pediatric intensive care unit; 7. Bronchoscopy; 9. Pediatric ward; 12. Radiology; 1212. Other
arr	Destination:<br>1. Neonatal Unit, intensive care; 2. Neonatal Unit, intermediate care; 3. Neonatal unit, specialized care; 4. Operating room; 5. Pediatric intensive care unit; 7. Bronchoscopy; 9. Pediatric ward; 12. Radiology; 1212. Other
dur_tri	Duration of transport (minutes)
nurse	Nurse present during transport: 1. Yes; 0. No
ass_nurse	Assistant nurse present during transport: 1. Yes; 0. No
resident	Resident present during transport: 1. Yes; 0. No
registrar	Registrar present during transport: 1. Yes; 0. No
other	Other staff present during transport: 1. Yes; 0. No
n_staff	Number of caregivers present during transport
vehicle	1. Incubator; 2. Radiant warmer; 3. Bed; 4. Stroller; 5. MR Diagnostics Incubator System Nomag®; 6. Other
resp	1. Invasive ventilation ; 2. Non invasive ventilation ; 3. Nasal canulae
no	Inhaled Nitric Oxide: 1. Yes; 0. No
any_catheter	Vascular access device: 1 = Yes; 0 = No
vvp	Number of peripheral venous catheters
vvp>0	Peripheral venous catheter: 1 = Yes; 0 = No
cvc	Central venous catheter: 1. Yes; 0. No
art_line	Arterial catheter: 1. Yes; 0. No
amines	Vasoactive drugs (continuous infusion): 1. Yes; 0. No

neurol	Sedative/analgesics (continuous infusion)
g_tube	Gastric tube: 1 = Yes; 0 = No
d_tube	Duodenal tube: 1 = Yes; 0 = No
gd_tube	Gastric or duodenal tube: 1 = Yes; 0 = No
bladder_c	Bladder catheter: 1 = Yes; 0 = No
omd	Other medical device: 1 = Yes; 0 = No
complication	Any adverse event (AE) during transport: 1 = Yes; 0 = No
Complicated_pat	Patient with at least 1 transport complicated by an AE: 1 = Yes; 0 = No
hypothermia	Hypothermia: 1 = Yes; 0 = No
hypothermie_spec	Hypothermia: specify temperature (degree Celsius)
hyperthermia	Hyperthermia: 1 = Yes; 0 = No
hyperthermie_spec	Hyperthermia: specify temperature (degree Celsius)
desat	Desaturation: 1 = Yes; 0 = No
desat_spec	Desaturation: specify minimal peripheral oxygen saturation
apnea	Apnea: 1 = Yes; 0 = No
other_resp_c	Other respiratory complication: 1 = Yes; 0 = No
other_resp_c_spec	Other respiratory complication: specify
agit	Agitation: 1 = Yes; 0 = No
pain	Pain: 1 = Yes; 0 = No
seiz	Seizures: 1 = Yes; 0 = No
other_neuro_c	Other neurological complication: 1 = Yes; 0 = No
bradyc	Bradycardia: 1 = Yes; 0 = No
bradyc_spec	Bradycardia: specify heart rate (beats/min)
tachyc	Tachycardia: 1 = Yes; 0 = No
tachyc_spec	Tachycardia: specify heart rate (beats/min)
hypotens	Agitation: 1 = Yes; 0 = No
hypotens_spec	Hypotension: specify mean arterial pressure (mmHg)
hypertens	Agitation: 1 = Yes; 0 = No
hypertens_spec	Hypertension: specify mean arterial pressure (mmHg)
other_cv_c	Other cardiovascular complication: 1 = Yes; 0 = No
other_cv_c_spec	Other cardiovascular complication: specify
equip_prob	Equipment problem: 1 = Yes; 0 = No
equip_prob_spec	Equipment problem: specify
med_error	Medication error: 1 = Yes; 0 = No
med_error_spec	Medication error: specify
other_c	Other adverse event: 1 = Yes; 0 = No
other_c_spec	Other adverse event: specify
intervention	Intervention (= modification of treatment during transport) : 1 = Yes; 0 = No
modif_med	Changes in dose of medication administered as continuous infusion: 1 = Yes; 0 = No
modif_med_spec	Change in dose of medication administered as continuous infusion: specify
admin_med	Medication (bolus): 1 = Yes; 0 = No
admin_med_spec	Medication (bolus): specify
bolus	Fluid bolus: 1 = Yes; 0 = No
remplissage_spec	Fluid bolus: specify
modif_resp	Change in respirators support: 1 = Yes; 0 = No
modif_fi	Change in FiO2: 1 = Yes; 0 = No
other_interv	Other intervention during transport: 1 = Yes; 0 = No
other_interv_spec	Other intervention during transport: specify

severity	Level of harm of adverse events (AE): 0 = no AE; 1 = no harm; 2 = mild harm; 3 = moderate harm; 4 = severe harm; 5 = death
temp_pre	Temperature before transport
temp_dur	Temperature during transport
temp_post	Temperature after transport
hr_pre	Heart rate before transport
hr_dur	Heart rate during transport
hr_post	Heart rate after transport
rr_pre	Respiratory rate before transport
rr_post	Respiratory rate after transport
sap_pre	Systolic arterial pressure before transport
sap_dur	Systolic arterial pressure during transport
sap_post	Systolic arterial pressure after transport
dap_pre	Diastolic arterial pressure before transport
dap_dur	Diastolic arterial pressure during transport
dap_post	Diastolic arterial pressure after transport
map_pre	Mean arterial pressure before transport
map_dur	Mean arterial pressure during transport
map_post	Mean arterial pressure after transport
sat_pre	Oxygen saturation before transport
sat_dur	Oxygen saturation during transport
sat_post	Oxygen saturation after transport
fi_pre	Fraction of inspired oxygen before transport
fi_dur	Fraction of inspired oxygen during transport
fi_post	Fraction of inspired oxygen after transport
